# Publisher Correction: Observation of transverse spin Nernst magnetoresistance induced by thermal spin current in ferromagnet/non-magnet bilayers

**DOI:** 10.1038/s41467-017-02303-8

**Published:** 2018-01-05

**Authors:** Dong-Jun Kim, Chul-Yeon Jeon, Jong-Guk Choi, Jae Wook Lee, Srivathsava Surabhi, Jong-Ryul Jeong, Kyung-Jin Lee, Byong-Guk Park

**Affiliations:** 1 Department of Materials Science and Engineering and KI for Nanocentury, KAIST, Daejeon, 34141 Korea; 2 Department of Materials Science and Engineering, Graduate School of Energy Science Technology, Chungnam National University, Daejeon, 34134 Korea; 3 Department of Materials Science and Engineering, Korea University, Seoul, 02841 Korea; 4 KU-KIST Graduate School of Converging Science and Technology, Korea University, Seoul, 02841 Korea

Correction to: *Nature Communications* 10.1038/s41467-017-01493-5, Article published online 9 November 2017

The original version of this Article contained an error in ref. ^27^, which was incorrectly given with the wrong journal name as:

Meyer, S. et al. Observation of the spin Nernst effect. *Nat. Phys*. **16**, 977–981 (2017).

The correct form of ref. ^27^ is:

Meyer, S. et al. Observation of the spin Nernst effect. *Nat. Mater*. **16**, 977–981 (2017).

This has now been corrected in the PDF and HTML versions of the Article.

