# Supplementary material for: Observation of transverse spin Nernst magnetoresistance induced by thermal spin current in ferromagnet/non-magnet bilayers
Source: Nat Commun. 2017 Nov 9;8:1400. doi: 10.1038/s41467-017-01493-5 (PMC5680200; doi:10.1038/s41467-017-01493-5)
Supplement: Supplementary file 1 — Supplementary Information [file 41467_2017_1493_MOESM1_ESM.pdf]

**Supplementary Note 1. Temperature distribution generated by laser illumination**

We estimated the temperature profiles of CoFeB(2 nm), W(3 nm)/CoFeB(2 nm) and Pt(3 nm)/CoFeB(2 nm) layers on the $SiO_2$(100 nm)/Si(650 μm) substrate using the heat transfer module of the COMSOL software. A monochromatic 660-nm continuous laser beam of 55 mW is considered to have Gaussian power density distribution. The function for the pulse is as follows

$$y(x) = \frac{1}{\sigma\sqrt{2\pi}} e^{\frac{-(x-x_0)^2}{2\sigma^2}}, \tag{1}$$

where $x_0$ is the centre location of the laser beam, $x$ is the distance from the centre, and $\sigma$ is the standard deviation. The power intensity of the laser decays exponentially from the beam centre in $z$-direction along the thickness of the samples. The Gaussian laser beam has full width half maximum of ~3$\sigma$, equivalent to its experimental diameter of 5μm. The finite element modeling in COMSOL software has been performed using the material parameters obtained under the same experimental conditions as the thermoelectric measurement. The absorption coefficients of W, Pt and CoFeB are $8.66\times10^5$ $cm^{-1}$, $7.78\times10^5$ $cm^{-1}$ and $8.9\times10^5$ $cm^{-1}$, respectively and the total reflectance of CoFeB(2 nm), W(3 nm)/CoFeB(2 nm), and Pt(3 nm)/CoFeB(2 nm) are measured as 0.119, 0.244, and 0.255, respectively. The temperature profiles were calculated utilizing the above parameters for the simulation area of 10 μm × 1 mm, of which size is equivalent to the experimented structure.

The absorbed electromagnetic wave in a thin film deposited on the $SiO_2$/Si substrate is evaluated by the absorption coefficients that is the characteristic feature of particular thickness of respective materials. In an ultra-thin film structure, absorption process could be affected by the multiple reflection and related interference effects, which is considered by solving the Maxwell's electromagnetic wave equations via finite difference time domain (FDTD) optical simulations.

When the laser illuminates on the centre of the sample, a vertical temperature difference $\Delta T_z$ of 54 mK, 54 mK, and 63 mK is generated along the single CoFeB, W/CoFeB, and Pt/CoFeB structures, respectively, while an lateral temperature difference $\Delta T_x$ over the sample structure cancels out, which are illustrated in Supplementary Figs. 1-3. Note that the capping layer of MgO(1 nm)/Ta(1 nm) is included in the calculation. In contrast, when the laser is moved away from the centre of the structure, net $\Delta T_x$ appears. Supplementary Figure 4 shows the lateral temperature differences along the *x*-direction ($\Delta T_x$) which were calculated by integrating temperature over the locally-excited area from $-2\sigma_{\mathrm{sd}}$ to $+2\sigma_{\mathrm{sd}}$ in the *y*-direction, where the thermal spin current is mostly converted into a transverse voltage. Here, $\sigma_{\mathrm{sd}}$ is the standard deviation of the temperature distribution. As a result, similar lateral temperature difference between left and right edge $\Delta T_x$ for both W/CoFeB and Pt/CoFeB samples is obtained to be ~24 K while the $\Delta T_x$ is ~17 K for CoFeB. When a laser locates at the edge of the sample, the effective length for temperature gradient and thermal voltage generation, $L_{\mathrm{T}}$ and $L_{\mathrm{V}}$ were defined by $2\sigma_{\mathrm{sd}}$ in *x*-direction and $4\sigma_{\mathrm{sd}}$ in *y*-direction, respectively.

Here we show that the COMSOL simulation result is reasonable by performing an additional measurement. First of all, we note that a typical RT curve measurement does not allow us to estimate the temperature gradient caused by the laser illumination by the following reason. The laser illumination thermally excites a local area where sample temperature is efficiently increased; however, the local heating makes it difficult to measure the sample temperature by measuring the resistance because the resistance increase of the illuminated area (~a few micron range) does not markedly contribute to the total resistance of the sample (~mm size).

In order to resolve the size difference issue between the sample and the laser spot, we fabricated a sample with a narrow wire structure as illustrated in Supplementary Fig. 5a. Note

that in this new sample, the resistance is dominated by the narrowest wire region of which width is even smaller than the laser spot size. We first measured the resistance of the sample as a function of the temperature in physical property measurement system (PPMS, Quantum design), showing a linear relation between the resistance and the sample temperature (Supplementary Fig. 5b). Then, we measured the variation of the resistance with a laser illumination. When the laser (55 mW) is on the wire, the resistance is increased from 1202 Ω to 1232 Ω as shown in Supplementary Fig. 5c, corresponding to the increase in temperature from 296 K to 324 K. On the other hand, when the laser is moved away from the wire of ~10 μm, the resistance is 1213 Ω, which corresponds to the temperature of 307 K. As a result, $\Delta T_x$ in the sample of 10 μm width is estimated to be 17 K, which is comparable to that obtained by simulation. Because of the non-identical sample structure, this experiment cannot determine the temperature profile in the real sample of Fig. 3; however, this confirms that the laser illumination can induce a temperature difference in the sample with a similar order of magnitude as the calculated value.

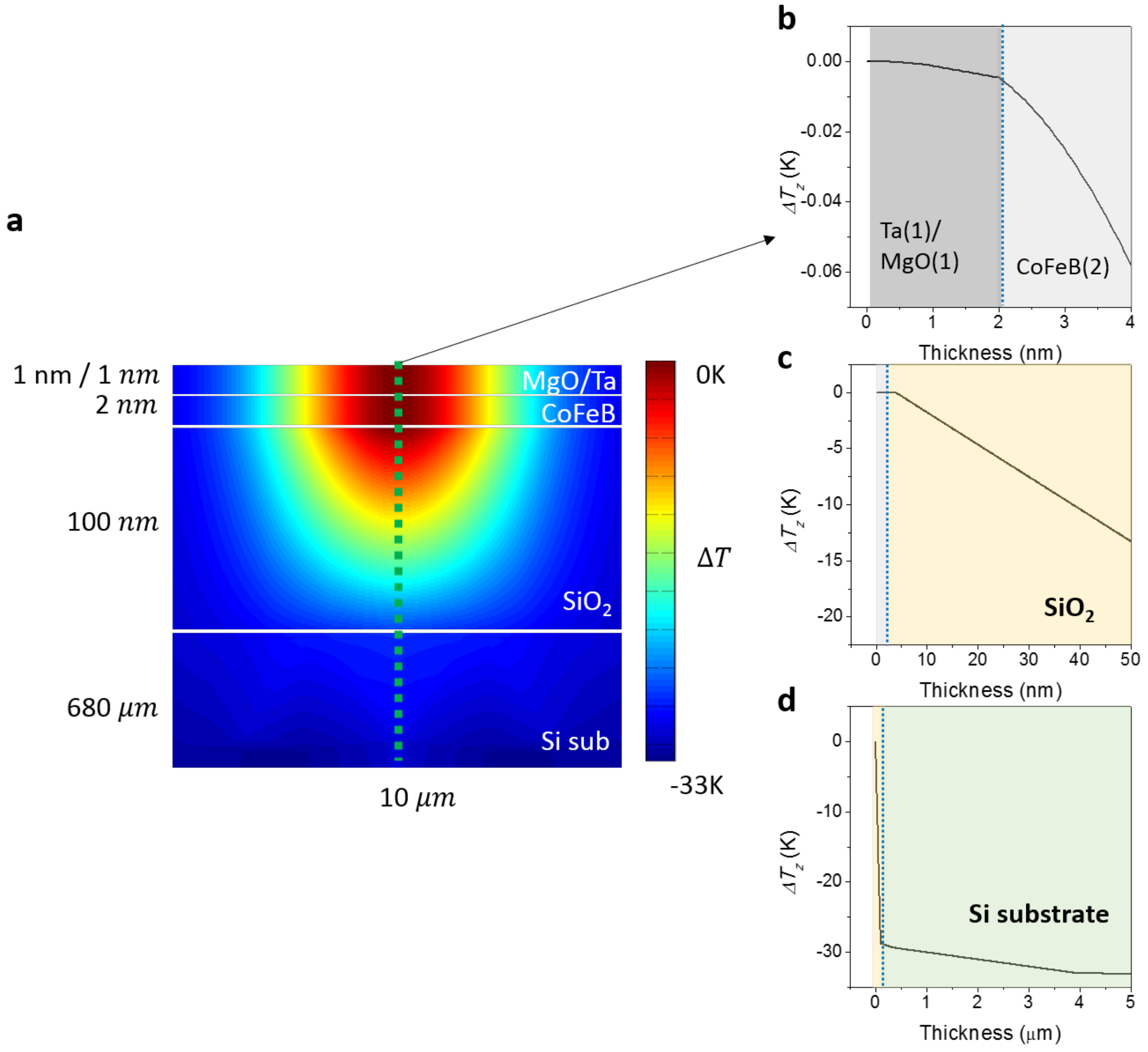


**Supplementary Figure 1 | Temperature profiles in Ta(1 nm)/MgO(1 nm)/CoFeB(2 nm)/$SiO_2$(100 nm)/Si substrate. a**, Cross-sectional view of temperature profile calculated under the illumination of a laser spot of 5 μm. **b-d,** Temperature profiles along the green-dotted line (vertical temperature distribution at laser centre) within the top Ta(1 nm)/MgO(1 nm)/CoFeB(2 nm) (b), to $SiO_2$(50 nm) (c), to Si substrate(5 μm) (d).

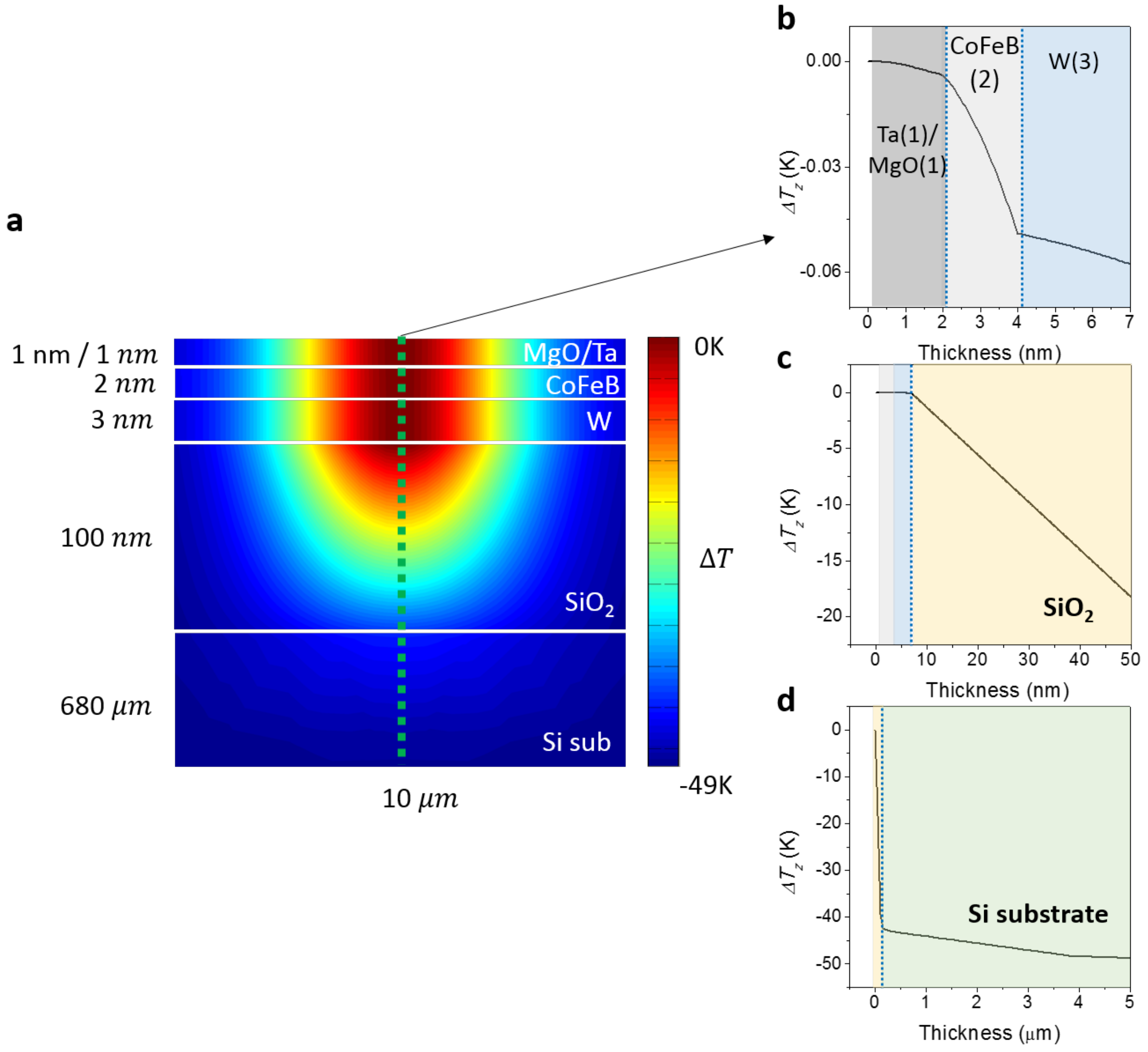


**Supplementary Figure 2 | Temperature profiles in Ta(1 nm)/MgO(1 nm)/W(3 nm)/CoFeB(2 nm)/$SiO_2$(100 nm)/Si substrate. a,** Cross-sectional view of temperature profile calculated under the illumination of a laser spot of 5 μm. **b-d,** Temperature profiles along the green-dotted line (vertical temperature distribution at laser centre) within the top Ta(1 nm)/MgO(1 nm)/W(3 nm)/CoFeB(2 nm) (b), to $SiO_2$(50 nm) (c), to Si substrate(5 μm) (d).

.

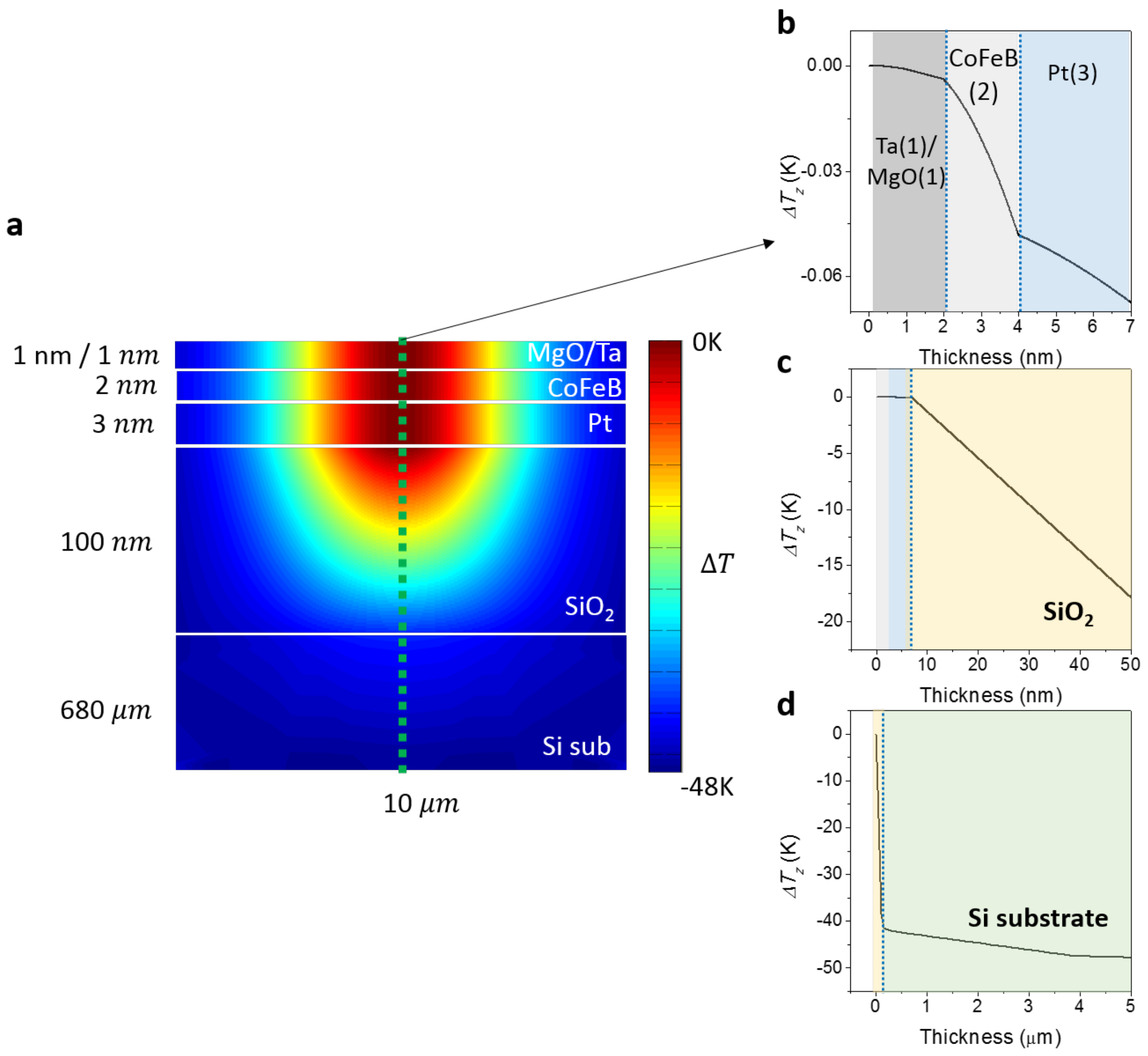


**Supplementary Figure 3 | Temperature profiles in Ta(1 nm)/MgO(1 nm)/Pt(3 nm)/CoFeB(2 nm)/$SiO_2$(100 nm)/Si substrate. a,** Cross-sectional view of temperature profile calculated under the illumination of a laser spot of 5 μm. **b-d,** Temperature profiles along the green-dotted line (vertical temperature distribution at laser centre) within the top Ta(1 nm)/MgO(1 nm)/Pt(3 nm)/CoFeB(2 nm) (b), to $SiO_2$(50 nm) (c), to Si substrate(5 μm) (d).

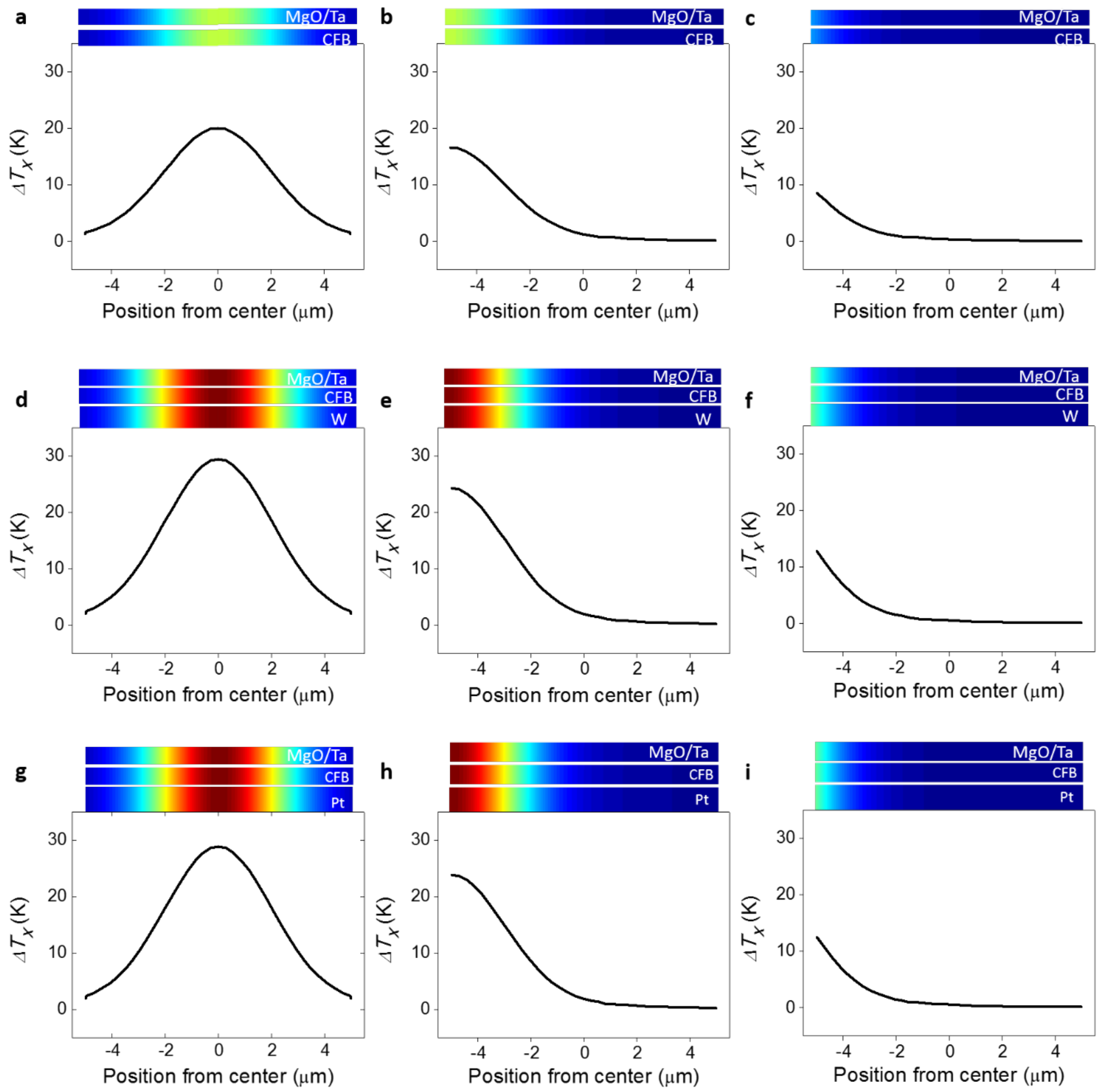


**Supplementary Figure 4 | Effective lateral temperature profiles at different laser locations.** Lateral temperature distribution for **a-c,** CoFeB(2 nm), **d-f,** W(3 nm)/CoFeB(2 nm) and **g-i,** Pt(3 nm)/CoFeB(2 nm) sample with capping layer of MgO(1 nm)/Ta(1 nm) at different laser locations. The laser is located on the centre of the sample ($x$=0 μm) (a, d, g), on the edge of the sample ($x$=5 μm) (b, e, h), and on the just outside of the sample ($x$=10 μm) (c, f, i).

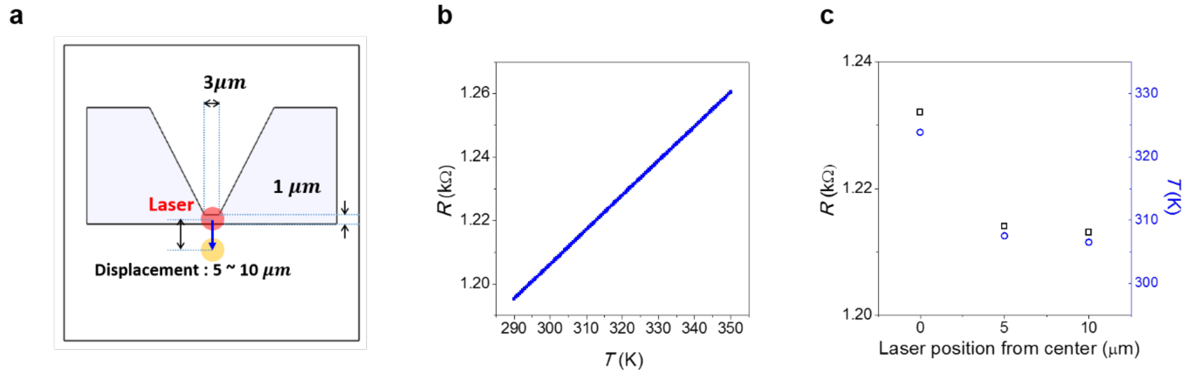


**Supplementary Figure 5 | Temperature variation by laser illumination. a,** Schematics of the sample structure with a wire of 1 μm width and 3 μm length. **b,** Temperature dependence of the resistance in Pt(4 nm)/CoFeB(2 nm) sample. **c,** Change in resistivity and resultant temperature by the laser illumination at different laser position from the centre of the sample.

**Supplementary Note 2. Magnetic field dependence of thermoelectric signal**

To verify the effect of magnetic field on the spin Nernst magnetoresistance (SNMR), we repeated the measurement for the W(3 nm)/CoFeB(2 nm) sample with different magnetic fields of 30 mT, 60 mT, and 100 mT. Supplementary Figure 6 demonstrates that the thermoelectric signals are almost independent of the magnetic field when it is large enough to saturate the magnetization to field direction.

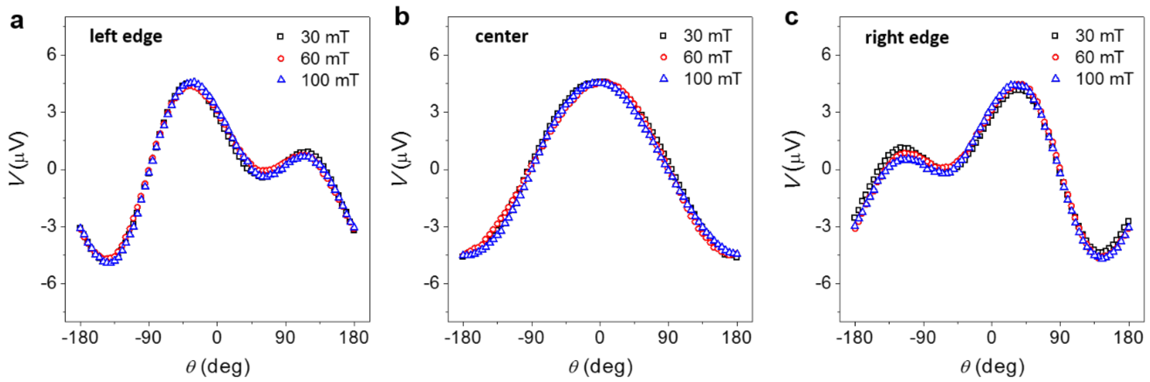


**Supplementary Figure 6 | Transverse spin Nernst magnetoresistance in W(3 nm)/CoFeB(2 nm). a-c**, Thermoelectric Hall signals for laser illumination under different magnetic fields of 30 mT, 60 mT, and 100 mT at left edge (a), centre (b), right edge (c) of the sample.

**Supplementary Note 3. Thermoelectric signals depending on laser position along the $y$-axis**

We perform the measurement of the thermoelectric Hall voltage (the same measurement as Fig. 2) with varying position along the $y$-axis (Supplementary Fig. 7a). We find that the transverse thermoelectric signals are almost identical for the measurements with different $y$-positions (Supplementary Fig. 7b-d), indicating that there is no considerable effect of the conventional Seebeck effect in our measurement configuration. This is attributed to the local excitation by the laser heating (diameter ~5 μm) in the elongated sample structure: 10 μm × 1 mm in which the $\Delta T_y$ between the two ends of the sample is not significantly generated by the laser illumination. This confirms the SNMR signal in our sample is mostly dominated by the $\Delta T_x$.

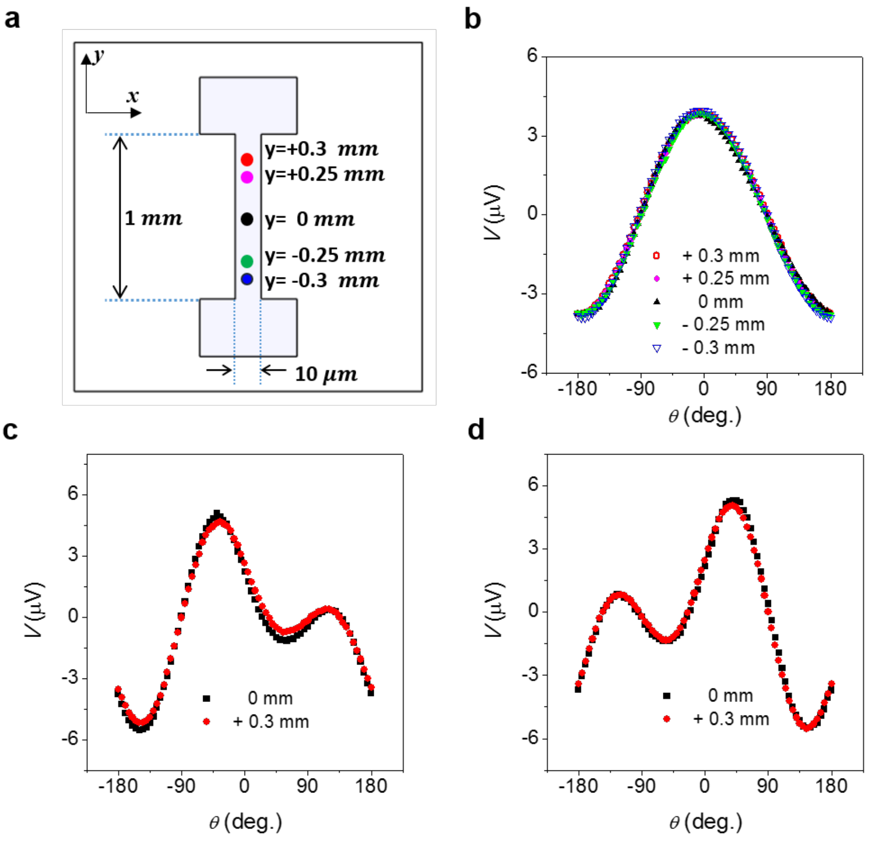


**Supplementary Figure 7 | Thermoelectric signals depending on laser position along the *y*-axis in W(3 nm)/CoFeB(2 nm) sample. a,** Schematics of the sample structure for the measurement. Each color represents a different laser location along the *y*-axis. **b,** Thermoelectric Hall signals under centre illumination with different laser locations. **c, d,** Thermoelectric Hall signals under the left edge (c) and the right edge (d) illumination of the laser located at $y = 0$ mm (black) and $y \sim +0.3$ mm (red).

**Supplementary Note 4. Planar Hall effect for W/CoFeB structure**

Supplementary Figure 8 shows the planar Hall effect (PHE) in CoFeB (2 nm) and W (3 nm)/CoFeB (2 nm) samples which are the same samples for the transverse spin Nernst magnetoresistance (SNMR) measurement shown in Fig. 2. The W/CoFeB sample shows a larger PHE signal than the CoFeB sample by a factor of ~20. In order to study the origin of the enhancement in the PHE, we investigated the angular dependence of the magnetoresistance in the W/CoFeB sample. We measured the longitudinal ($R_{xx}$) and transverse ($R_{xy}$) resistance in W($t_W$)/CoFeB(2 nm) samples with $t_W$=2, 4, and 5 nm while rotating the sample on three major planes of the *x*-*y*, *y*-*z*, and *z*-*x* planes under a magnetic field of 9 T. The angle of each plane is denoted as $\alpha$, $\beta$, and $\gamma$, respectively, as indicated in Supplementary Fig. 9. The $\Delta R_{xx}$ represents the spin Hall magnetoresistance (SMR), anisotropic magnetoresistance (AMR), and a sum of the SMR and AMR for the $\beta$-scan, $\gamma$-scan, and $\alpha$-scan, respectively. Supplementary Figures 9-11 show that the SMR is much more dominant than the AMR in the W/CoFeB sample. Moreover, the $\Delta R_{xy}$ with $\alpha$ is attributed to the PHE which is the same amount as the $\Delta R_{xx}$, demonstrating the enhancement in the PHE is mostly contributed by the transverse SMR. Supplementary Figure 12 shows the W thickness dependence of the SMR, which is consistent with that of the SNMR.

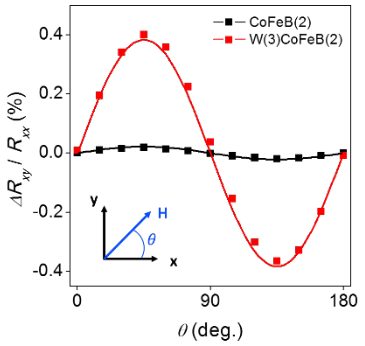


**Supplementary Figure 8 | Planar Hall effect in CoFeB(2 nm) and W(3 nm)/CoFeB(2 nm) samples.** The PHE signal ($\Delta R_{xy}/R_{xx}$) for CoFeB (black) and W/CoFeB (red) is plotted as a function of in-plane angle on $x$-$y$ plane. Black and red curves represent $sin2\theta$ fitting curves.

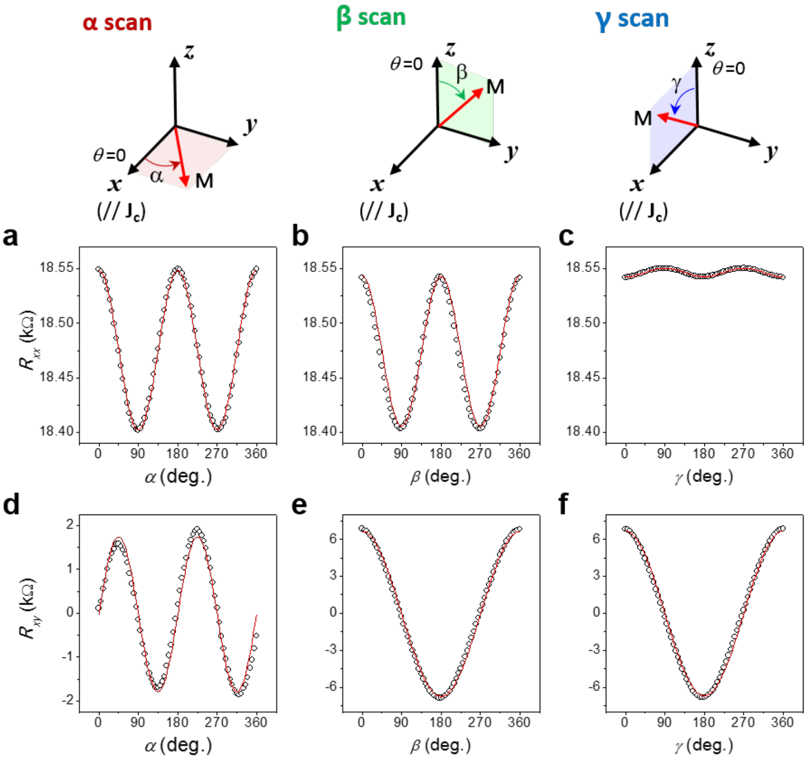


**Supplementary Figure 9 | Angular dependence of the magnetoresistance in W(2 nm)/CoFeB(2 nm) sample.** Angular dependence of the longitudinal resistance $R_{xx}$ (**a, b, c**) and transverse resistance $R_{xy}$ (**d, e, f**) of the W(2 nm)/CoFeB(2 nm) sample for $\alpha$, $\beta$, and $\gamma$ scan, respectively. The measurements were done under a magnetic field of 9 T.

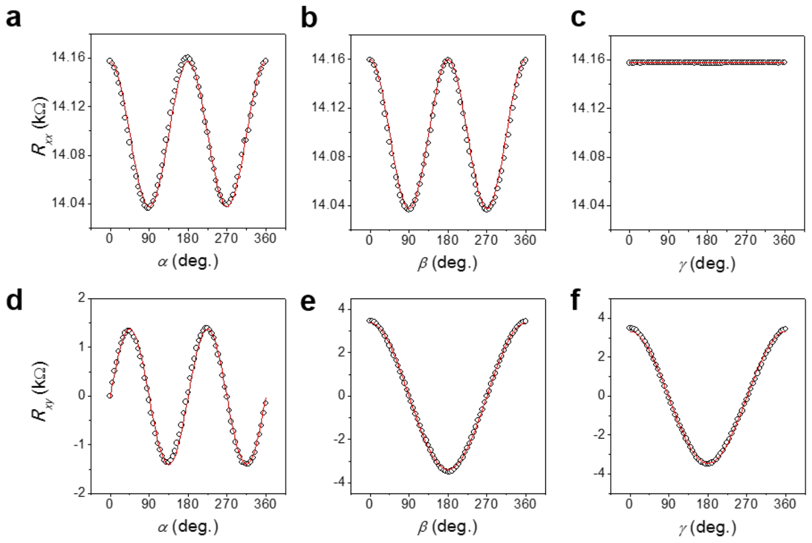


**Supplementary Figure 10 | Angular dependence of the magnetoresistance in W(4 nm)/CoFeB(2 nm) sample.** Angular dependence of the longitudinal resistance $R_{xx}$ (**a, b, c**) and transverse resistance $R_{xy}$ (**d, e, f**) of the W(4 nm)/CoFeB(2 nm) sample for $\alpha$, $\beta$, and $\gamma$ scan, respectively.

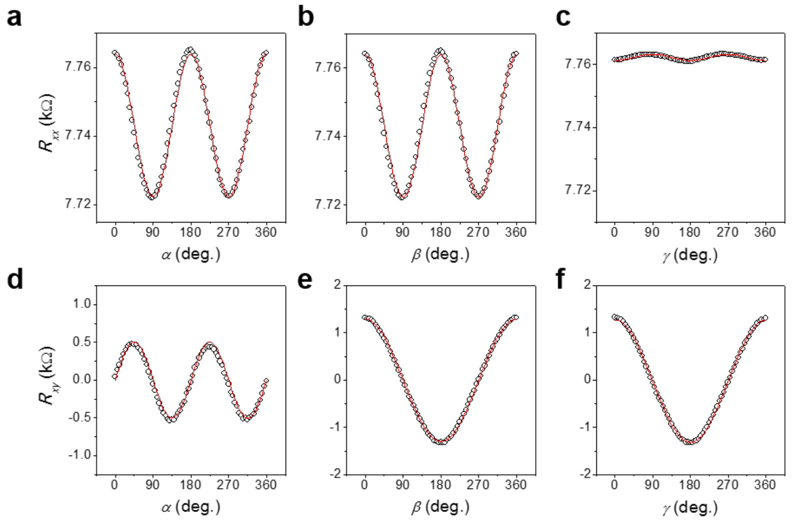


**Supplementary Figure 11 | Angular dependence of the magnetoresistance in W(5 nm)/CoFeB(2 nm) sample.** Angular dependence of the longitudinal resistance $R_{xx}$ (**a, b, c**) and transverse resistance $R_{xy}$ (**d, e, f**) of the W(5 nm)/CoFeB(2 nm) sample for $\alpha$, $\beta$, and $\gamma$ scan, respectively.

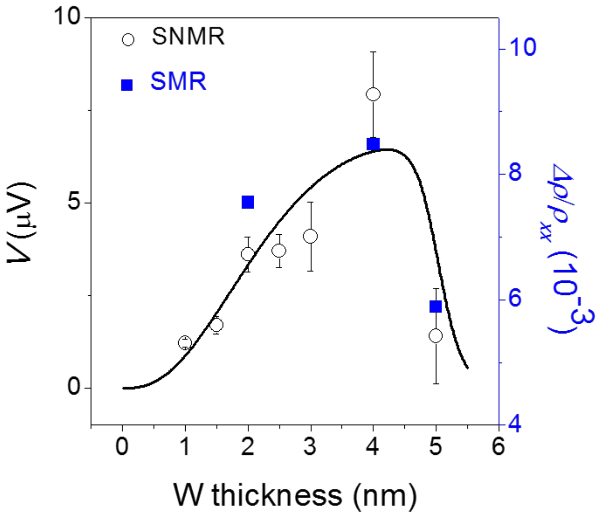


**Supplementary Figure 12 | Comparison between transverse SNMR and SMR in W/CoFeB.** W thickness dependence of the Transverse SNMR and SMR in W(1~5 nm)/CoFeB(2 nm). Error bars represent the range of the measured values, resulting from laser position uncertainty.

**Supplementary Note 5. Decomposition of thermoelectric Hall signals in HM/CoFeB**

We studied the dependence of the HM thickness on the thermoelectric Hall signal in W($t_W$)/CoFeB and Pt($t_{Pt}$)/CoFeB structures. Supplementary Figure 13 shows the angular dependence of the thermoelectric Hall voltages for each sample at various laser locations. As thermoelectric Hall voltages consist of $cos\theta$ and $sin2\theta$ components ($V_\theta$, $V_{2\theta}$), which are related to $\Delta T_z$ and $\Delta T_x$, respectively, we decomposed them by fitting the measured data with Equation (S2).

$$V = V_\theta \cos(\theta) + V_{2\theta} \sin(2\theta) + C, \qquad \ldots \text{(S2)}$$

where $C$ is a d.c. offset of the thermoelectric Hall voltage which depends on the sample or measurement configuration. The offset is simply subtracted when the SNMR is analyzed in Fig. 2 and Supplementary Fig. 13 because it does not have an angular dependence that is a characteristic feature of the SNMR. The extracted $V_\theta$ as a function of laser position from the centre of the sample in W/CoFeB and Pt/CoFeB are shown in Supplementary Fig. 14, while the $V_{2\theta}$ components are presented in Fig. 3a,b. The $V_\theta$ in all samples decreases as the laser position moves away from the centre of the sample which is explained by the reduction of the heating area on the structure, or smaller $\nabla T_z$. This indicates that the $V_\theta$ mainly originated from the spin Seebeck effect and anomalous Nernst effect which are proportional to $\nabla T_z$ [1,2].

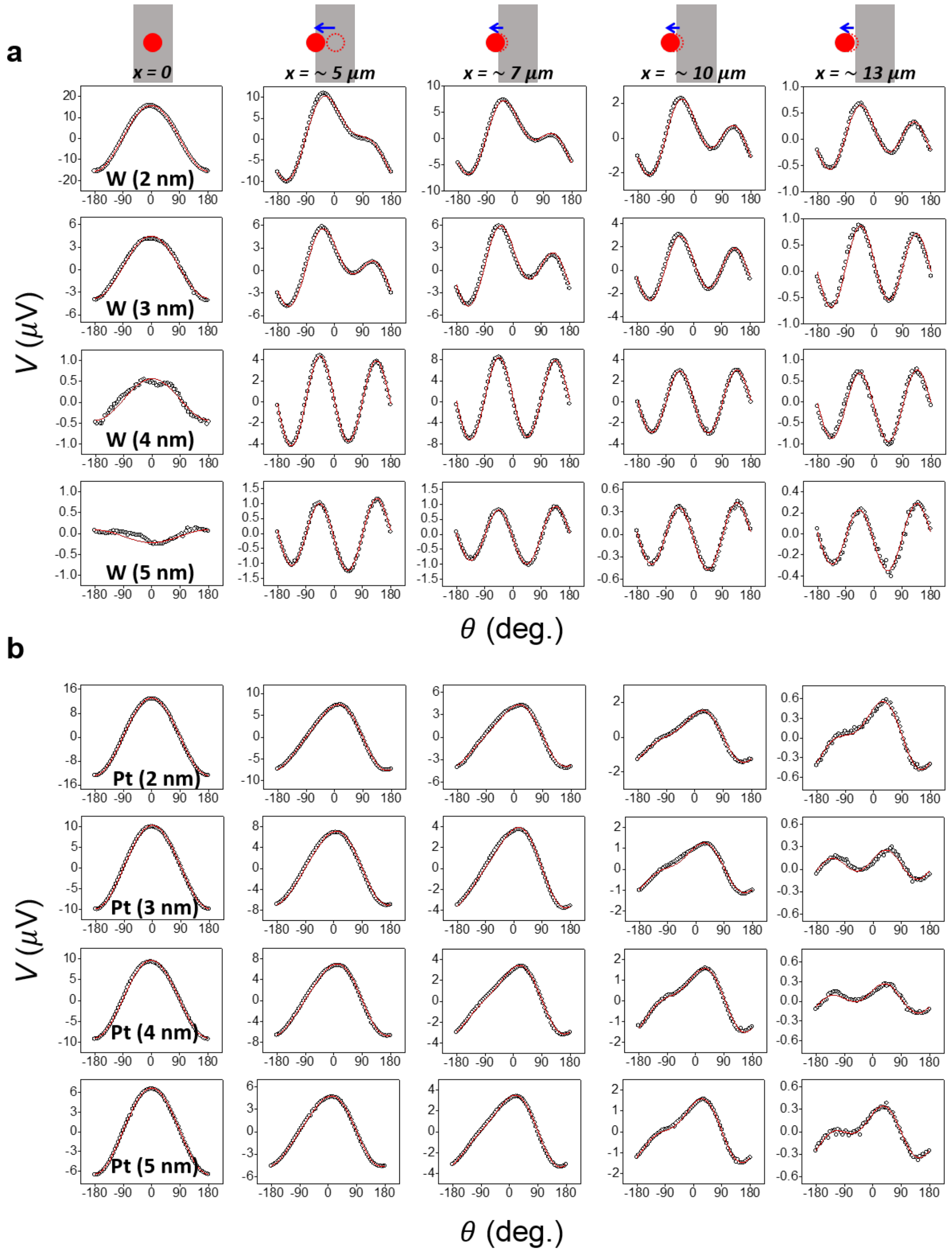


**Supplementary Figure 13 | Thermoelectric Hall signals in W/CoFeB and Pt/CoFeB samples. a, b**, Thermoelectric Hall signals for W($t_W$)/CoFeB(2 nm) with $t_W$=2~5 nm (a) and Pt($t_{Pt}$)/CoFeB(2 nm) with $t_{Pt}$=2~5 nm (b). The red lines are fitting curves using Supplementary Equation (1). Top schematics illustrate the laser position of each measurement.

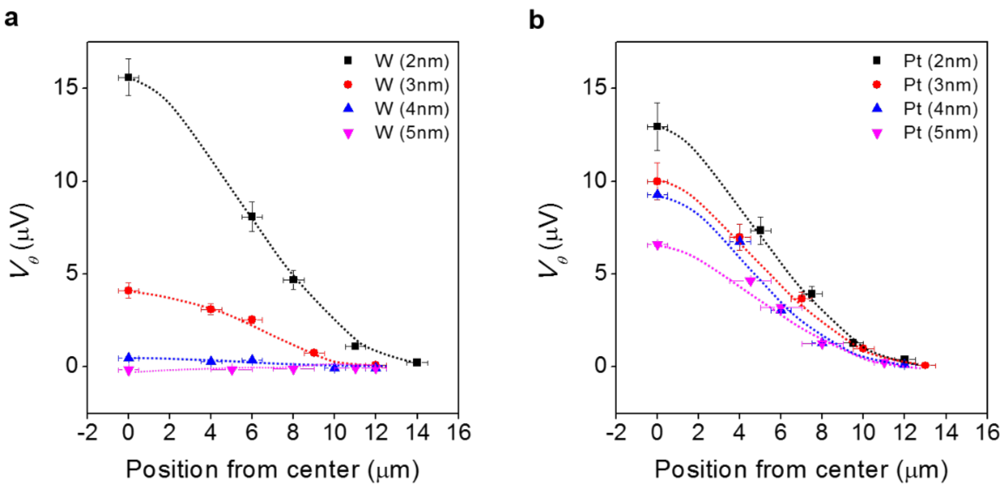


**Supplementary Figure 14 | Laser position dependence of the thermoelectric Hall signals.** **a, b**, The extracted $V_\theta$ as a function of distance of the laser position from the centre of the sample for W/CoFeB (a) and Pt/CoFeB structures (b). Error bars represent the range of the measured values, resulting from laser position uncertainty.

**Supplementary Note 6. Thickness dependence of the resistivity in W**

The resistivity of W varies with its thickness. We measured the resistance of the W/CoFeB samples as a function of W thickness, demonstrating that the resistivity starts to decrease for the W thickness larger than 4 nm (see Supplementary Fig. 15a). This is attributed to the phase change in W; from β-W to α-W as the thickness increases [3]. The variation of the resistivity alters the geometric factor $\chi_{\mathrm{HM}}$ as well (see Supplementary Fig. 15b). The resistance and $\chi_{\mathrm{HM}}$ variations in W/CoFeB samples were taken into account when the spin Nernst angle was estimated in the main text.

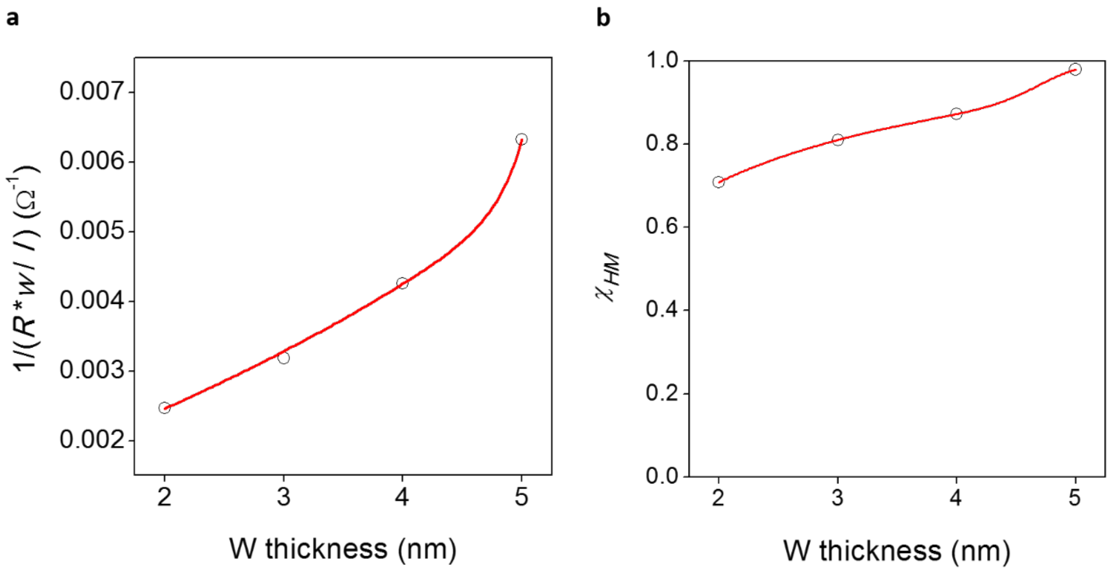


**Supplementary Figure 15 | W thickness dependence of resistivity and geometric factor. a,** Reciprocal of resistance in W electrode as a function of its thickness in W/CoFeB (2 nm) structures, which is normalized by sample geometry: the line width *w* and length *l*. **b,** Geometric factor $\chi_{\mathrm{HM}}$ variation as a function of W thickness.

**Supplementary References**


[1] Kim, D.-J. *et al*. Utilization of the antiferromagnetic IrMn electrode in spin thermoelectric devices and their beneficial hybrid for thermopiles. *Adv. Funct. Mater.* **26**, 5507-5514 (2016).

[2] Lee, K.-D. *et al*. Thermoelectric signal enhancement by reconciling the spin Seebeck and anomalous Nernst effects in ferromagnet/non-magnet multilayers. *Sci. Rep.* **5**, 10249 (2015).

[3] Cho, S., Beak, S. C., Lee, K.-D., Jo, Y. & Park, B.-G. Large spin Hall magnetoresistance and its correlation to the spin-orbit torque in W/CoFeB/MgO structures. *Sci. Rep.* **5**, 14668 (2015).
